# hiPSC-derived NSCs effectively promote the functional recovery of acute spinal cord injury in mice

**DOI:** 10.1186/s13287-021-02217-9

**Published:** 2021-03-11

**Authors:** Desheng Kong, Baofeng Feng, Asiamah Ernest Amponsah, Jingjing He, Ruiyun Guo, Boxin Liu, Xiaofeng Du, Xin Liu, Shuhan Zhang, Fei Lv, Jun Ma, Huixian Cui

**Affiliations:** 1 Hebei Medical University-National University of Ireland Galway Stem Cell Research Center, Hebei Medical University, Shijiazhuang, 050017 Hebei Province China; 2 Hebei Research Center for Stem Cell Medical Translational Engineering, Shijiazhuang, 050017 Hebei Province China; 3 Human Anatomy Department, Hebei Medical University, Shijiazhuang, 050017 Hebei Province China

**Keywords:** Spinal cord injury, Induced pluripotent stem cell, Neural stem cell, Mesenchymal stem cell

## Abstract

**Background:**

Spinal cord injury (SCI) is a common disease that results in motor and sensory disorders and even lifelong paralysis. The transplantation of stem cells, such as embryonic stem cells (ESCs), induced pluripotent stem cells (iPSCs), mesenchymal stem cells (MSCs), or subsequently generated stem/progenitor cells, is predicted to be a promising treatment for SCI. In this study, we aimed to investigate effect of human iPSC-derived neural stem cells (hiPSC-NSCs) and umbilical cord-derived MSCs (huMSCs) in a mouse model of acute SCI.

**Methods:**

Acute SCI mice model were established and were randomly treated as phosphate-buffered saline (PBS) (control group), repaired with 1 × 10^5^ hiPSC-NSCs (NSC group), and 1 × 10^5^ huMSCs (MSC group), respectively, in a total of 54 mice (*n* = 18 each). Hind limb motor function was evaluated in open-field tests using the Basso Mouse Scale (BMS) at days post-operation (dpo) 1, 3, 5, and 7 after spinal cord injury, and weekly thereafter. Spinal cord and serum samples were harvested at dpo 7, 14, and 21. Haematoxylin-eosin (H&E) staining and Masson staining were used to evaluate the morphological changes and fibrosis area. The differentiation of the transplanted cells in vivo was evaluated with immunohistochemical staining.

**Results:**

The hiPSC-NSC-treated group presented a significantly smaller glial fibrillary acidic protein (GFAP) positive area than MSC-treated mice at all time points. Additionally, MSC-transplanted mice had a similar GFAP+ area to mice receiving PBS. At dpo 14, the immunostained hiPSC-NSCs were positive for SRY-related high-mobility-group (HMG)-box protein-2 (SOX2). Furthermore, the transplanted hiPSC-NSCs differentiated into GFAP-positive astrocytes and beta-III tubulin-positive neurons, whereas the transplanted huMSCs differentiated into GFAP-positive astrocytes. In addition, hiPSC-NSC transplantation reduced fibrosis formation and the inflammation level. Compared with the control or huMSC transplanted group, the group with transplantation of hiPSC-NSCs exhibited significantly improved behaviours, particularly limb coordination.

**Conclusions:**

HiPSC-NSCs promote functional recovery in mice with acute SCI by replacing missing neurons and attenuating fibrosis, glial scar formation, and inflammation.

**Graphical abstract:**

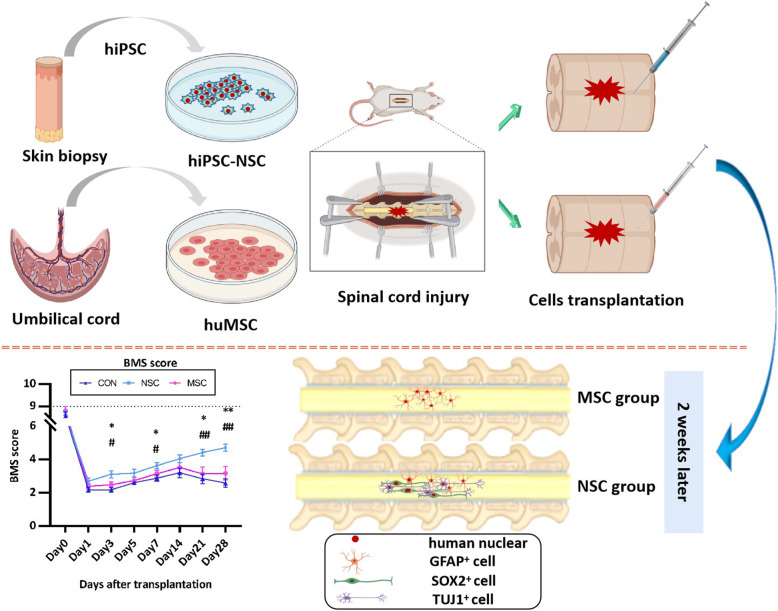

## Background

Spinal cord injury (SCI) is a common condition that potentially results in the devastating and permanent loss of neurological function due to the failure of axonal regeneration after injury, thereby interrupting the connection between the brain and the body. Based on the available evidence, the incidence of SCI has increased dramatically during the past decade in China, primarily due to an increase in spinal trauma caused by motor vehicle crashes and falls [1]. The Global Burden of Diseases, Injuries, and Risk Factors Study reported 0.93 million (95% uncertainty interval [UI] 0.78–1.16 million) new cases of SCI, and SCI caused 9.5 million (95% UI 6.7–12.4 million) years of life lived with disability in 2016 [2, 3]. Due to the lack of a gold standard or effective treatment, the real challenge facing scientists is identifying an appropriate therapy.

According to numerous preclinical studies, stem cell therapy holds great promise for the treatment of central nervous system (CNS) injuries. The administration of stem cells after traumatic SCI reduces neuronal loss and improves functional recovery in animal models [4–7]. Two desirable objectives of cell-based therapies are to induce trophic reactions (such as the production of extracellular matrix and diffusible growth factors) and to replace cells lost through injury or disease with transplant-derived cells (such as new oligodendrocytes and neurons) that will enhance the regenerative responses of the host’s CNS. Trophic factors secreted from transplanted cells have been shown to support neuronal survival and neurite outgrowth.

Mesenchymal stem cells (MSCs) have also attracted attention in cell-based therapy because they are easy to isolate and preserve in primary cultures, raise no ethical concerns and are unlikely to develop into tumours. Additionally, cross-reactivity is minimal or absent [8]. MSCs contribute to reducing the size of the spinal cord injury cavity in rat models of both mild and severe SCI while differentiating into glial fibrillary acidic protein (GFAP)-positive astrocytes and neuronal cells [9]. MSCs have also been shown to differentiate into oligodendrocytes but not into cells expressing neuronal markers [neuronal nuclei (NeuN)] [10]. Based on these results, MSCs may have therapeutic limitations in some respects.

While, neural stem cells (NSCs) hold promising therapeutic potential for the treatment of SCI. The NSCs could be harvested from foetal brain and spinal cord tissues, and also be derived from embryonic stem cells (ESCs) and induced pluripotent stem cells (iPSCs). The pioneering studies suggested that NSCs survived and differentiated into both neurons and glial cells within the lesion site, attenuated oxidative stress, promoted angiogenesis, integrated into synaptic relays, and enhanced nerve regeneration via the expression of neurotrophic factors and promotion of axonal growth and remyelination [11, 12]. However, less attention has been focused on the potential of transplanted NSCs to modulate localized and systemic inflammatory processes, fibrosis, and astrocytic activation, all of which are integral processes in SCI pathogenesis. Thus far, the generation of NSCs derived from iPSCs (iPSC-NSCs) not only addresses the limitations in terms of ethical concerns and availability but further offer the possibility of autologous transplantation when compared with NSCs harvested from ESCs or foetal tissue. Several research groups have done pioneering studies examining the transplantation of iPSC-NSCs for treatment of SCI, which have had promising results, with most of studies targeting subacute and chronic SCI [13–19]. The previous publications of human iPSC-NSCs (hiPSC-NSCs) in SCI have focused on axonal regrowth [19, 20], tumour development [21], and synapse formation [18, 20].

Furthermore, few studies have compared the efficacy of NSC and MSC treatments but in subacute SCI models in which the NSCs and MSCs are transplanted at least 7 days post-SCI injury [22, 23]. Therefore, in this study, we transplanted MSCs from the human umbilical cords (huMSCs) or hiPSC-NSCs into an SCI mouse model to examine the potential treatment-recovery effects in acute phase, and the inflammatory reactive changes around the injured regions and spread within the whole body, as well as the formation of fibrotic and glial scar.

## Materials and methods

### Ethics statement

The collection of all human samples and the performance of animal studies were approved by the Committee of Ethics on Experimentation of Hebei Medical University (assurance no. 20190505). The experiments were conducted in accordance with the National Institutes of Health Guidelines for the Care and Use of Laboratory Animals.

### Harvesting iPSC-derived NSCs

The hiPSCs were generated from dermal fibroblasts using a protocol previously described in a study from our lab (cell line: HEBHMUi002-A) [24]. Briefly, fibroblasts at passage 4 were reprogrammed into hiPSCs using the Cytotune™-iPS 2.0 Sendai Reprogramming kit (Thermo Fisher Scientific, Waltham, MA, USA) according to the manufacturer’s guideline. On day 0 of transduction, 4 reprogramming factors were added to fibroblasts, and the medium was changed 24 h later to remove the virus. On day 7 post-transduction, the cells were transferred to a 6-well plate coated with Geltrex hESC-qualified Basement Membrane Matrix (Thermo Fisher Scientific, Waltham, MA, USA) at the recommended density. After 24 h, the medium was changed to Essential 8™ Medium (Gibco, Grand Island, NY, USA); hiPSC colonies were ready to be manually picked an average of 14 days after transduction and characterized using standard validated methods.

We used the PSC Neural Induction Medium (Gibco) to induce iPSCs to become NSCs according to the recommended protocol. Briefly, iPSCs were cultured in the PSC neural induction medium [Neurobasal® Medium (Gibco) supplemented with 10% Neural Induction Supplement (Gibco)] on a Geltrex-coated 6-well plate, and the medium was changed every other day. After 7 days, the iPSCs differentiated into neural stem cells [24, 25]. The derived cells were fixed with 4% paraformaldehyde (Sigma, St. Louis, MO, USA) in DPBS (Invitrogen, Carlsbad, CA, USA) for immunofluorescence staining [Nestin, SOX2, Paired box 6 (PAX6), and Octamer binding transcription factor 4 (OCT4): Abcam, Cambridge, UK, detail information in Additional file 1: Table. S1].

### huMSC culture and characteristics

Human umbilical cords were obtained from full-term neonates after caesarean section at the Second Hospital of Hebei Medical University (Shijiazhuang, China). The patients had been informed a priori and had consented to donate the umbilical cords. The huMSCs were obtained by tissue mass culture. Briefly, under sterile conditions, the cord was cut into 3–4-cm pieces and placed on a dish containing saline solution; each section was cut along the umbilical vein and the outer membrane, venous and arterial walls were gently removed. The residual tissue was cut into 1-mm^3^ slices with scissors and fixed evenly at bottom of the culture dish. Complete MSC growth medium with supplement (Jingmeng, Beijing, China) was added to each dish at a volume that covered the bottom of the dish with a thin layer, and placed in a 37 °C, 5% CO_2_ incubator. Seventeen days later, cell growth was observed; the cells were passaged when they reached approximately 90% confluence. Passage 3–5 cells were used to detect the levels of the surface antigens cluster of differentiation (CD) 29, CD44, CD73, CD90, CD105, CD 45, and HLA-DR with flow cytometry. Fat induction, bone formation, and chondrocyte differentiation experiments were performed with the appropriate differentiation medium (Cyagen, Suzhou, China). Oil Red O staining, Alizarin Red staining, and Safranin O staining were used for specific staining.

### Establishment of the SCI model and stem cell transplantation

Eight-week-old female immunodeficient BALB/c nude mice (19–22 g, *n* = 54) were anaesthetized with an intraperitoneal injection of 1% pentobarbital sodium (50 mg/kg, i.p. Hubei XinRunde Chemical Co., Ltd., Wuhan, China). A laminectomy was performed at the 10th thoracic vertebra to expose the dorsal surface of the spinal dura mater. A contusive SCI was induced at the level of T10 using a Zhongshi impactor with weight of 10 g and height of 5 cm (Zhongshi, Beijing, China). In the NSC group, 1 × 10^5^ hiPSC-NSCs in 1 μl PBS were transplanted into the injured spinal cord (1 mm rostral to the lesion epicentre) of 18 mice using a glass pipette at a rate of 0.5 μl/minute with a 10-μl Hamilton syringe and a stereotaxic microinjector (RWD, Shenzhen, China). Using the same method, eighteen mice were injected with 1 × 10^5^ huMSCs in 1 μl PBS (MSC group), and 18 mice were injected with 1 μl of PBS (control group). All surgeries were conducted under anaesthesia, and efforts were made to minimize the animals’ suffering, with selected humane endpoints.

### Behavioural test of locomotor function

Six mice were selected from each treated group at random for behavioural tests at days post-operation (dpo) 1, 3, 5, 7, 14, 21, and 28. Hind limb motor function was evaluated in open-field tests using the Basso Mouse Scale (BMS) [26]. Mice were placed on a flat surface with dimensions of 20 cm × 40 cm and observed for 3 min. Two observers who were blinded to the groups simultaneously evaluated the animals’ behaviours, including coordination, trunk instability, and stepping. A score of 0 represents flaccid paralysis; a score of 9 represents normal gait. In brief, when only one or two assessable passes occurred, we scored the mouse as having “no coordination” regardless of whether coordination was demonstrated on those passes. Consequently, we classified coordination as none (none or 3 assessable passes), some (< 50% of assessable passes) and most (≥ 50% of assessable passes). Severe trunk instability occurs in two ways: the hindquarters show severe postural deficits such as extreme lean, pronounced waddle and/or near collapse predominantly during the test, or any of the haunch hits, spasms, scoliosis events that stop stepping of one or both hind limbs. Mild trunk instability is scored when there are five or more instances of mild trunk instability, fewer than five events that stop stepping or when severe trunk instability instances are not predominant during the test. If fewer than five instances of mild trunk instability occur during the testing period, normal trunk stability is scored. For the stepping score, 0 indicates no stepping, and scores of 1, 2, and 3 indicate occasional, frequent and consistent dorsal stepping with no plantar stepping, respectively. Scores of 4, 5, and 6 represent occasional, frequent, and consistent plantar stepping, respectively.

### Histology and immunofluorescence staining

Six mice were selected randomly from each treated group and sacrificed at time point dpo 7, 14, and 28. Mice were terminally anaesthetized, perfused with saline, and then perfused with 4% paraformaldehyde (PFA) in PBS for fixation. The spinal cords were removed from the body, maintained overnight in 4% PFA (4 °C) then cryoprotected overnight in 30% sucrose in PBS. On the next day, the spinal cords were trimmed to the injured portion (5 mm of the total length), embedded in Tissue-Tek compound (Wetzlar, Germany), frozen and maintained at − 80 °C. Tissue sections with a thickness of 14 μm were obtained for haematoxylin-eosin staining (H&E) and Masson’s trichrome staining.

A primary antibody directed against human nuclei (HuNu, human nuclei antibody, Gene Tex, Irvine, CA, USA) was used to identify human cells transplanted into the mouse spinal cord. Antibodies against GFAP (Thermo Fisher Scientific), beta III Tubulin (TUJ-1, Abcam, Cambridge, MA, UK), and SOX2 (Abcam, Cambridge, MA, UK) were used to follow the fate of the transplanted stem cells and their interaction with the host tissue. Frozen sections were permeabilized in 0.1% Triton X-100 for 20 min. Non-specific protein binding was blocked by incubating the sections with 5% goat serum in PBS for 30 min, followed by an incubation with the primary antibody solution. After an overnight incubation at 4 °C, the sections were successively washed twice with 0.05% PBS-Tween followed by PBS for 5 min each. Sections were then incubated with a DyLight 594-conjugated secondary goat anti-mouse IgG (H+L) antibody and FITC-conjugated goat anti-rabbit IgG (H+L) antibody (Thermo Fisher Scientific) for 1 h at room temperature. Nuclei were counterstained with 4,6-diamidino-2-phenylindole (DAPI, Cell Signalling, Danvers, MA, USA).

### Measurement of serum cytokine levels

The serum levels of cytokines were measured to assess the effects of the transplantation of various stem cells (huMSCs and hiPSC-NSC) on inflammation at 7, 14, and 28 days after SCI (*n* = 6 animals from each group per time point). The blood was collected, incubated overnight at 4 °C, and centrifuged at 5000 rpm, after which the sera were collected for ELISAs to measure the cytokines. ELISAs for VEGF, IL-6, and TNF-α were performed using mouse ELISA kits (Abconal, Wuhan, China) according to the manufacturer’s instructions. The OD value at 450 nm (reference at 570 nm) was detected with SPARK (Tecan Trading AG, Switzerland), and the absolute concentration was calculated from the standard curve.

### Quantitative analysis

The outlines of the lesion area were drawn manually from the images of longitudinal sections at the epicentre of the injury and the area was calculated using Image J (version 1.50i, National Institute of Health, USA) [27]. For inflammatory cell count, we used 5 digital photographs of a 400 × 400-pixel square of the HE-stained specimens taken near the epicentre and counted inflammatory cells manually with Image J (version 1.50i, National Institute of Health, USA) [28]. Four high-power (× 200) fields in each section stained with Masson’s trichrome were selected to calculate the ratio of the fibrotic area of the spinal cord to the whole area using Image-Pro Plus 6.0 software (Media Cybernetics, Silver Spring, MD, USA) [29]. The intensity of immunofluorescence staining for GFAP was quantified by measuring the integrated optical density (IOD) using Image-Pro Plus 6.0 software, as described in a previous report [30]. Briefly, glial scar area was quantified by measuring the area of dense GFAP staining near the injury epicentre and excluding the lesion, as defined by the absence of GFAP staining, using a 150-μm grid. The average GFAP staining intensity was obtained.

### Statistical analysis

All quantitative data are reported as the means ± standard errors of the means (SEM) and were analysed using the SPSS 22.0 (StatSoft, Tulsa, OK, USA) or GraphPad PRISM 7.0 (GraphPad Software, San Diego, USA) statistical software. The statistical significance of differences between the three groups was evaluated using one-way ANOVA, while Tukey’s multiple comparisons test was used as a post hoc analysis to assess differences between any two groups. Statistical significance was set to *p* < 0.05.

## Results

### The characterization of hiPSC-NSCs and huMSCs

**Fig. 1:**
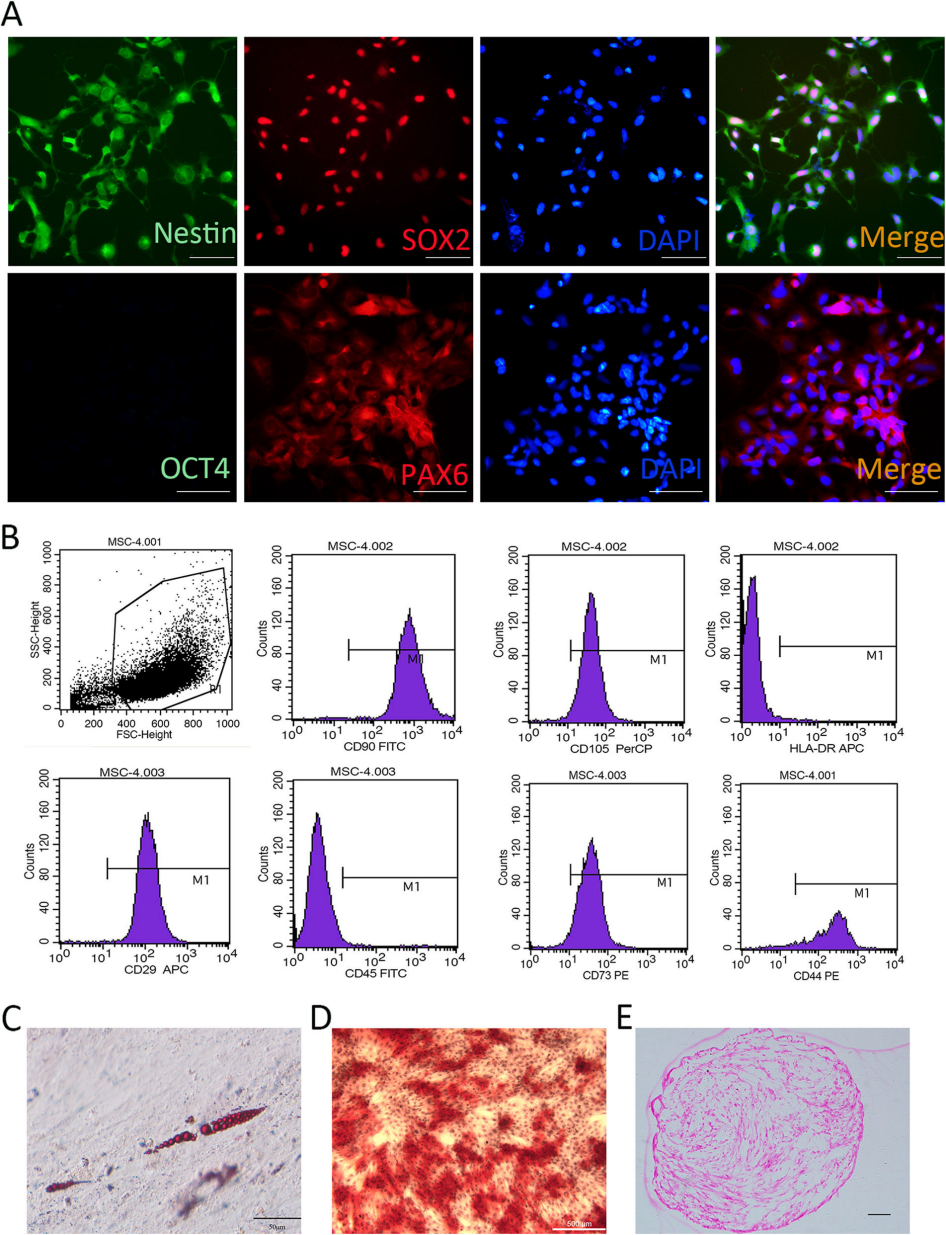
The characteristic of hiPSC-NSC and huMSC. **a** The hiPSC-NSC were positive for nestin, SOX2 and PAX6. **b** Cell surface antigens were detected at passage 4 using flow cytometry. huMSCs were positive for CD29, CD44, CD73, CD90, and CD105. In contrast, very little expression of the haematopoietic lineage markers HLA-DR and CD45 was detected in huMSCs. **c** Oil red O staining of lipid droplets revealed that huMSCs differentiated into cells of the adipogenic lineage. Bar, 50 μm. **d** Alizarin red staining revealed a calcified extracellular matrix of induced huMSCs, confirming that huMSCs differentiated into osteogenic cells. Bar, 500 μm. **e** Safranin O staining showed the secretion of the extracellular matrix of chondrocytes

The hiPSC-NSCs that were derived from hiPSCs expressed the markers Nestin, SOX2, and PAX6 (Fig. 1a). Flow cytometry and induced differentiation were performed to characterize the huMSCs. As shown in Fig. 1b, the huMSCs were positive for specific MSC surface markers, including CD29, CD44, CD73, CD90, and CD105, and negative for CD45 and HLA-DR (Fig. 1b). The huMSCs were capable of adipogenic, osteogenic, and chondrogenic differentiation as determined by the results of Oil Red O, Alizarin Red S, and Alcian Blue staining, respectively (Fig. 1c–e).

### Pathomorphological changes

**Fig. 2:**
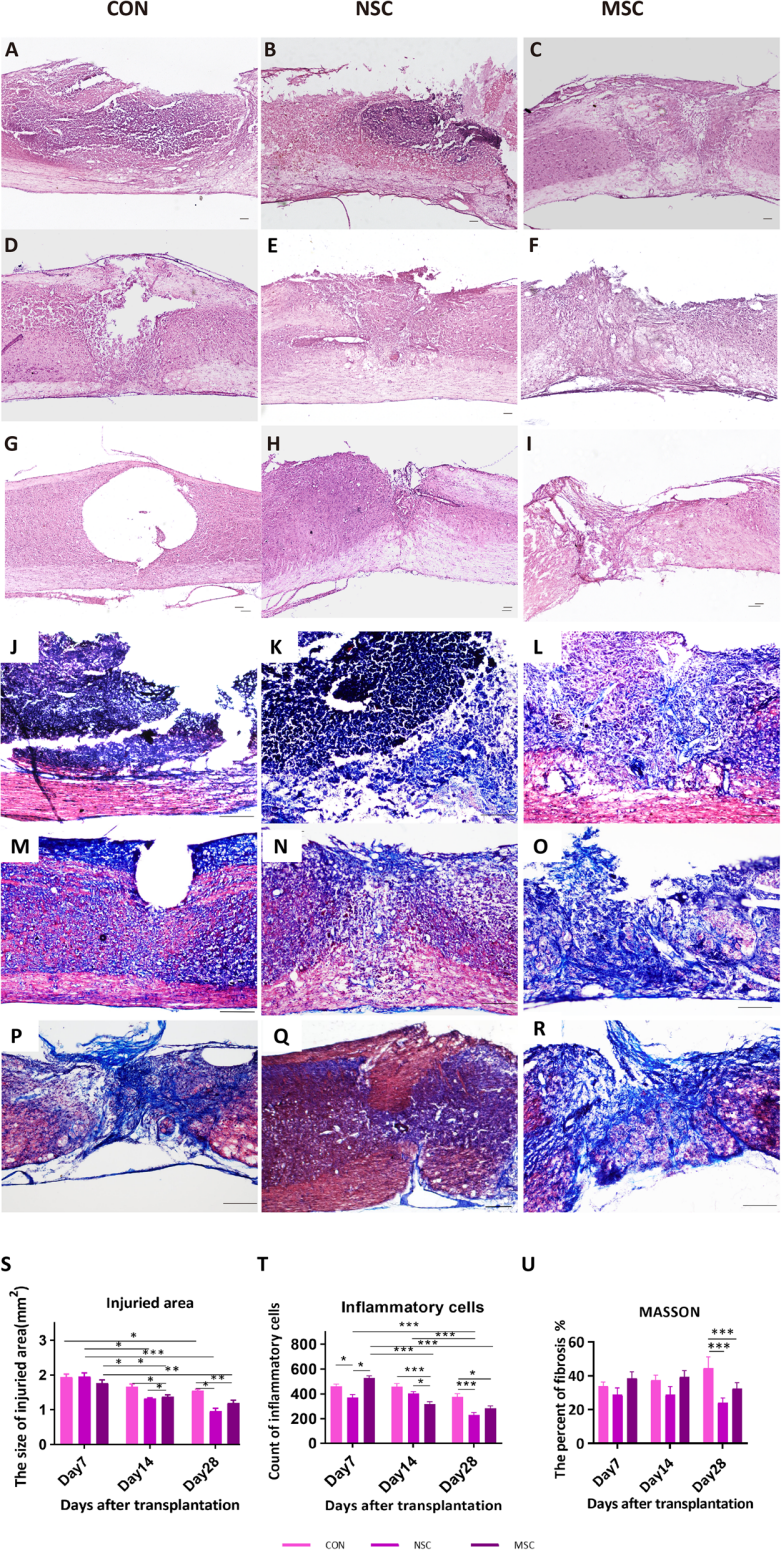
The pathomorphological changes. Images of HE staining and Masson’s trichrome staining in the different groups at dpo 7(**a**–**c**, **j**–**l**), dpo 14 (**d**–**f**, **m**–**o**), and dpo 28 (**g**–**i**, **p**–**r**). The injured area sizes in three groups at dpo 28 were significantly decreased compared to those at dpo 7, and the sizes in NSC group and MSC group were significantly lower than that in the control group at dpo 28 (**s**). The MSC and NSC groups exhibited a significantly lower level of inflammatory cell infiltration compared to the control group at dpo 28. The percentage of fibrosis in the NSC and MSC groups was significantly lower compared to the control group (**u**). Scale bar, 100 μm. *p < 0.05; **p < 0.01; ***p < 0.001

A cavity was formed in the injured spinal cord of the control group, and the surface of the injured spinal cord was collapsed. In contrast, the surface of the injured spinal cord in the NSC group was plump, smooth, and not significantly collapsed (Fig. 2). At dpo 28, numerous inflammatory cells had infiltrated the SCI site in the control group. The MSC group exhibited a reduced level of inflammatory cell infiltration compared to the control group (282.9 ± 20.99 vs. 377.2 ± 25.23, *p* < 0.05), and a reduced area of necrotic tissue (1.20 ± 0.08 mm^2^ vs. 1.55 ± 0.05 mm^2^, *p* < 0.01). The SCI site of the NSC group spanned a smaller area than that of the control group (0.96 ± 0.09 mm^2^ vs. 1.55 ± 0.05 mm^2^, *p* < 0.05) (Fig. 2s, t). The percentage of fibrosis at the SCI site was evaluated to assess recovery and scar formation. At all time points, the smallest fibrotic area was observed in the NSC group among all three groups, although the differences were not significant at dpo 7 and dpo 14. A significantly lower average percentage of fibrosis was observed in the NSC group compared to control group (23.33 ± 2.03% vs. 64.48 ± 3.10%, *p* < 0.001) and the percentage of fibrosis was significantly decreased in the MSC group compared to the control group (32.51 ± 3.51% vs. 64.48 ± 3.10%, *p* < 0.001).

### Astrogliosis following stem cell transplantation

**Fig. 3:**
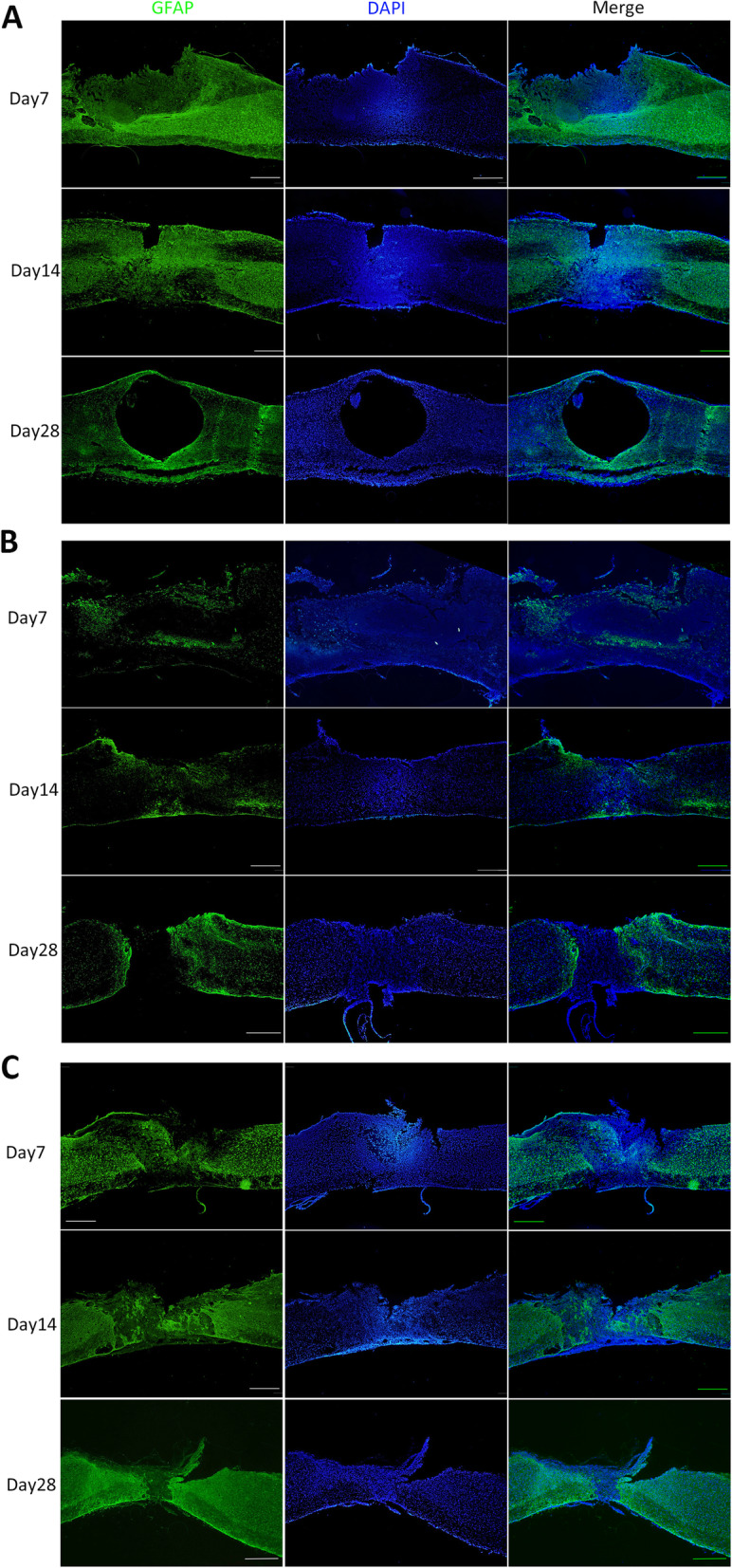
Longitudinal spinal cord sections (14 μm) were immunostained for GFAP to detect astrogliosis. Scale bar, 500 μm

**Fig. 4:**
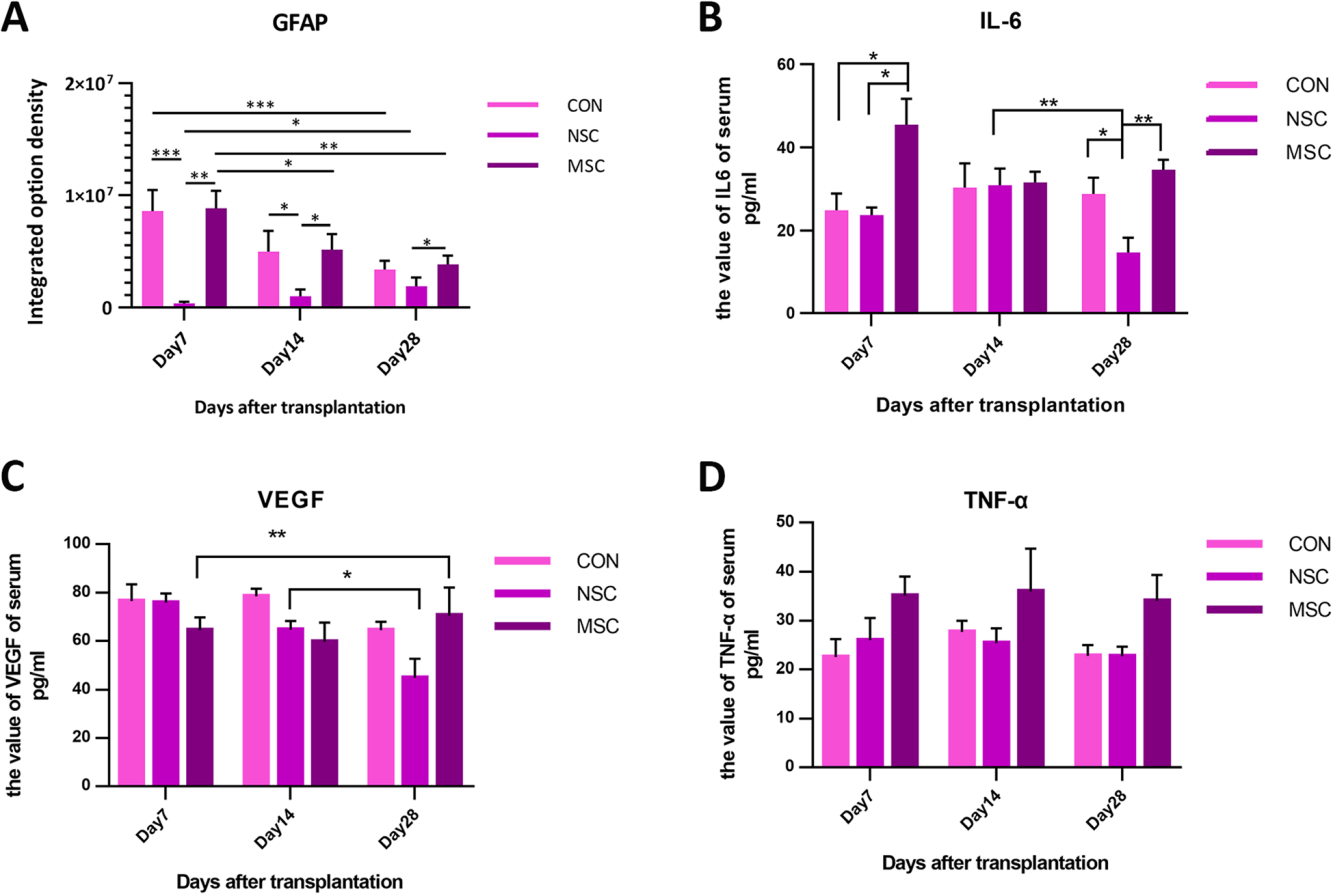
The GFAP expression level in the spinal cord injury site (**a**) and the serum IL-6 (**b**), VEGF (**c**), and TNF-α (**d**) levels at dpo 7, 14, and 21. All data are presented as the means ± SEM: significance was determined using ANOVA followed by Tukey’s post hoc test. *p < 0.05; **p < 0.01; ***p < 0.001

Anti-GFAP immunostaining was performed to assess the distribution of astrocytes and formation of a glial scar at dpo 7, 14, and 28 after contusion (Fig. 3a–c). Astrocytes were packed tightly and formed a scar barrier in the control group. However, in the NSC group, astrocytes appeared to be permissive and did not form prominent glial limitans to completely block regenerating axons (Fig. 3b). As expected, the lesions in the NSC group exhibited significantly reduced GFAP-positive glial scarring. In contrast, a massive glial scar was observed in the MSC group that was not significantly different from the control group (Fig. 3). Quantitative analysis revealed a significant decrease in the IOD of GFAP in the NSC group compared to the control and MSC groups at dpo 7 and 14. Moreover, the IOD of GFAP showed a decreasing trend over time in the control and MSC groups [control group: dpo 7 (8.59 × 10^6^) vs. dpo 28 (3.42 × 10^6^), *p* < 0.001; MSC group: dpo 7 (8.84 × 10^6^) vs. dpo 28 (3.87 × 10^6^), *p* < 0.001], while its IOD was slightly increased over time in the NSC group, but these differences were not significantly [dpo 7 (3.69 × 10^5^), dpo 14 (9.98 × 10^5^), dpo 28 (1.89 × 10^6^)]. The hiPSC-NSC-treated group presented a significantly reduced IOD of GFAP compared to the MSC-treated group at all time points. Additionally, MSC-transplanted mice exhibited a similar IOD of GFAP in mice receiving PBS (Fig. 4a).

### The fate and directional differentiation of transplanted cells

**Fig. 5:**
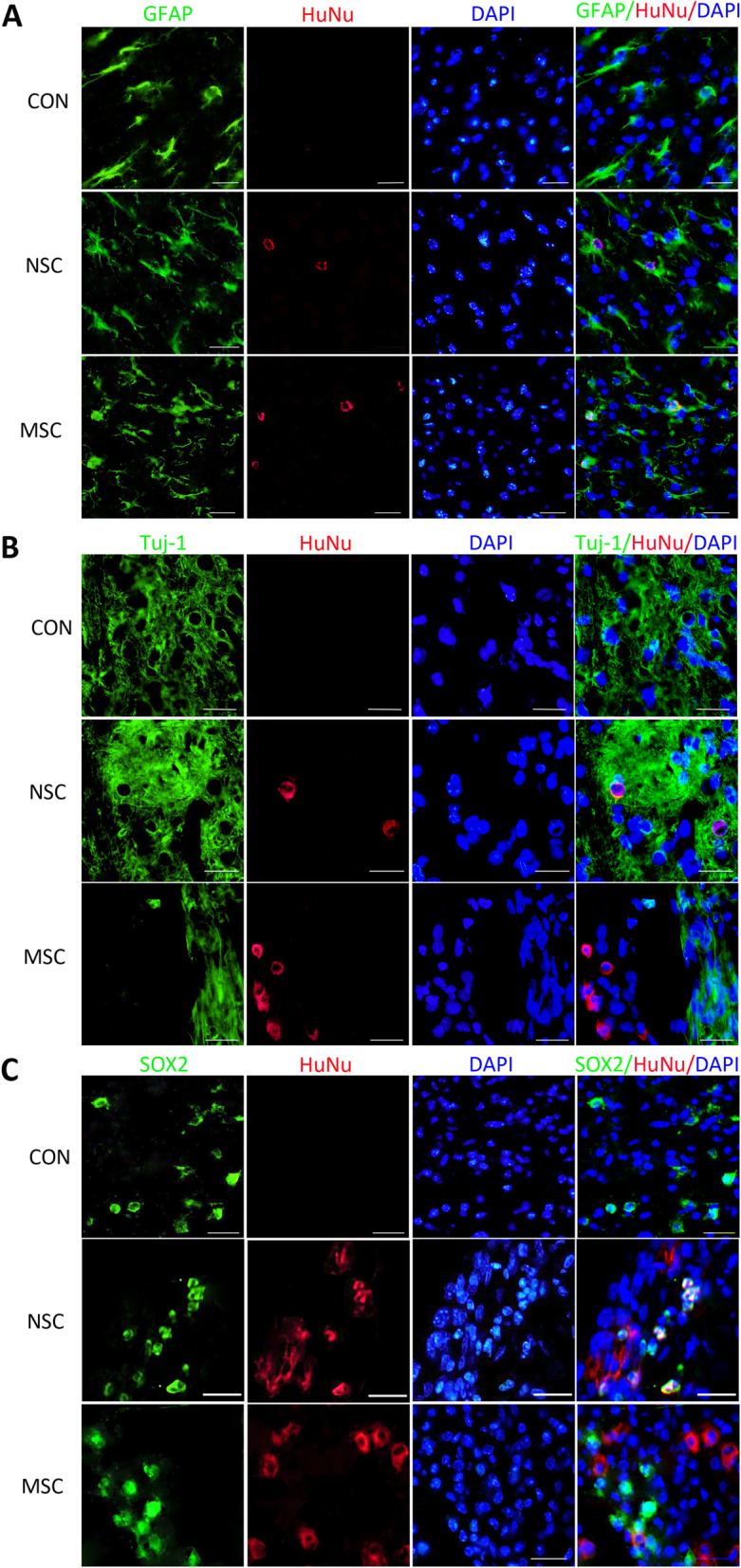
The survival and differentiation of transplanted stem cells. Fluorescence images of immunofluorescence staining for the GFAP, TUJ-1, and SOX2 markers at dpo 14. Scale bar, 25 μm

The surviving stem cells were stained with HuNu (Fig. 5a–c). Differentiation of the transplanted stem cells in the injured spinal cord of the three stem cell transplanted groups was identified using cell marker antibodies, and the control group was used as the negative control group. Cells with HuNu-positive nuclei (red) were observed in the spinal cord sections from the NSC group, and they were double stained with either GFAP or TUJ1 (Fig. 5). Thus, the transplanted cells had differentiated into astrocytes and neurons. Some cells with SOX2-positive nuclei were also observed, indicating that some NSCs were still alive (SOX2-positive cells). Cells with HuNu-positive nuclei (red) were observed in the MSC group and were double stained with the anti-GFAP antibody (green) but were not double stained with the anti-TUJ1 or anti-SOX2 antibody. In our experiment, the surviving huMSCs in the injured spinal cord differentiated into astrocytes, but not neural cells. No transplanted stem cells were observed in the control group.

### The serum cytokine levels in mice with a spinal cord injury

The level of IL-6 in the MSC group at dpo 7 was 45.50 ± 6.26 pg/ml, which was significantly higher than in the other groups (NSC group, 23.63 ± 1.80 pg/ml; control group, 25.57 ± 3.33 pg/ml). At dpo 14, no differences were observed among the three groups. At dpo 21, the IL-6 level was significantly decreased compared with the level detected at dpo 14 in the NSC group (13.56 ± 2.77 pg/ml), and a lower level was observed compared to the levels in the other groups at the same time point (Fig. 4b). VEGF levels in the NSC group decreased gradually as the injury healed. Significantly lower VEGF levels were observed in the NSC group than in the other two groups at dpo 28. In the MSC group, the VEGF level at dpo 28 was significantly higher than at dpo 7. The levels detected in PBS-treated animals remained unchanged at all time points (Fig. 4c). No significant difference in TNF-α levels was detected among three groups at different time points (Fig. 4d).

### Behavioural analysis of locomotor function

**Fig. 6:**
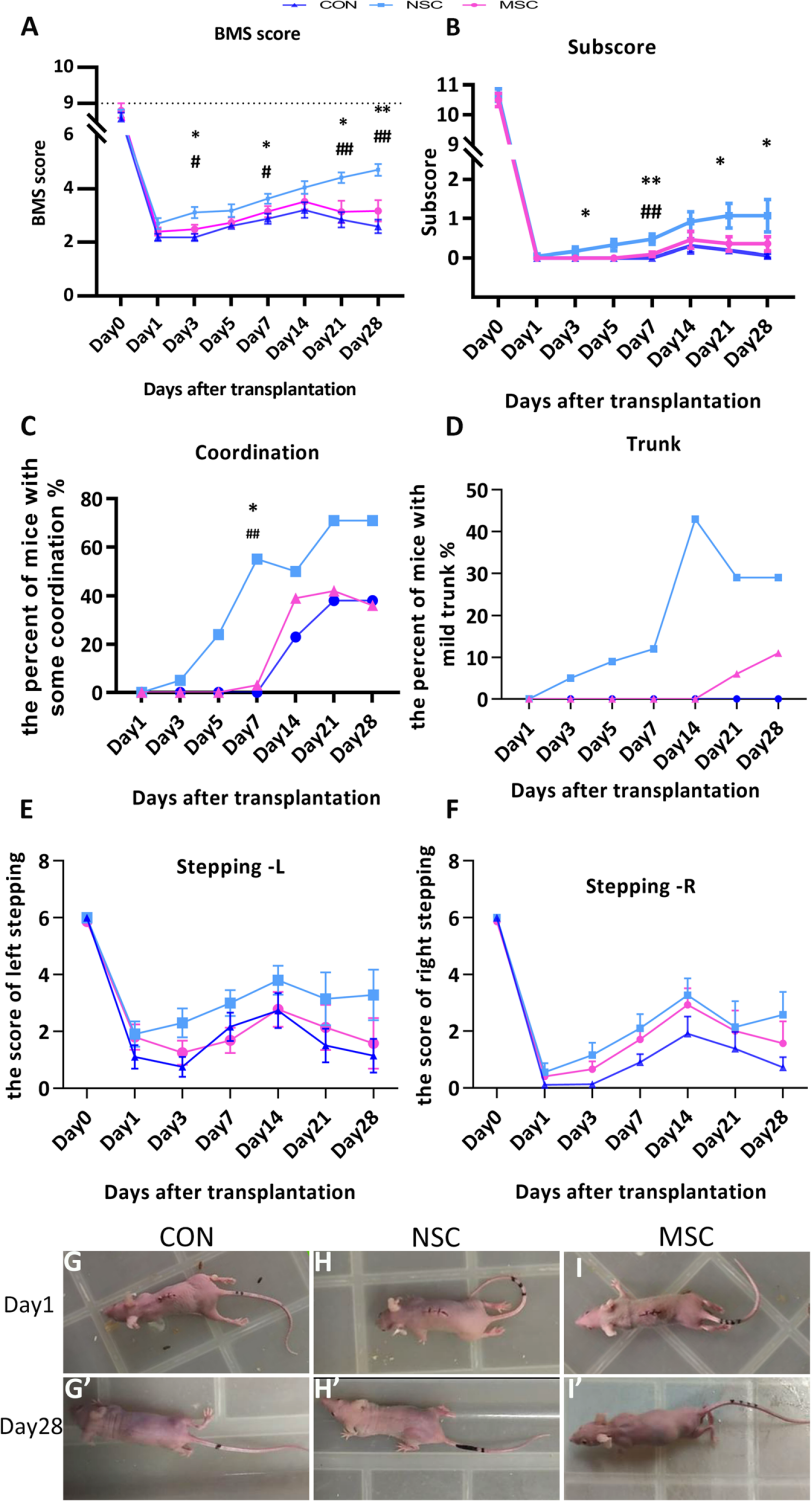
The recovery of locomotor function following stem cell transplantation after SCI. The locomotor function of PBS- or stem cell-treated mice was assessed using the BMS test: BMS score (**a**), subscore (**b**), coordination (**c**), trunk (**d**), and the stepping score of right side (**e**) and left side (**f**). All animals in the NSC group showed significantly higher performance in the open-field BMS test than MSC-treated animals and PBS controls (**a**). Significantly higher subscores were recorded for the NSC group than the other treatment groups. Additionally, the MSC-transplanted animals did not show better performance than controls (**b**). Regarding coordination (**c**), for which body weight-supported stepping is essential, a stable but insignificant trend of higher performance was observed in the NSC group. The trunk function in the NSC group recovered earlier and to a greater extent than the MSC and control groups (**d**). A stable but insignificant trend of higher performance on stepping function was observed in the NSC group after the grafting of stem cells (**e**, **f**). **g**–**i**, Mouse limb function at dpo 1; **g**′–**i**′, mouse limb function at dpo 28. *p < 0.05 compared with the control group; ^#^*p* < 0.05 compared with the MSC group

The huMSCs, hiPSC-NSC, or PBS in the control group were transplanted by an intramedullary injection into the injured area immediately after spinal cord contusion. After contusion-induced SCI, locomotor function was evaluated with the BMS locomotor rating scale in an open field. Almost complete hind limb paralysis was observed in both groups at dpo 1 (Fig. 6g–i, Additional files 2, 3, 4: Videos 1–3), and BMS scores of the three groups were similar (Fig. 6a). The locomotor function gradually improved in the NSC group throughout the observation period, while the function of the MSC group and control group recovered before dpo 14 and then decreased. At dpo 3, the score of NSC group was significantly higher than the scores of the other two groups (3.11 ± 0.21 vs. 2.49 ± 0.17 and 2.19 ± 0.14). Similarly, a higher BMS score was recorded for the NSC group at dpo 7 and 14. Interestingly, transplanted animals in the NSC group always showed better maximum scores on the BMS test at shorter times after SCI. At dpo 28, the muscles of the lower limbs and buttocks of mice in the control group and MSC group had obviously atrophied, while the shapes of muscles were approximately normal in the NSC group (Fig. 6g′–i′, Additional files 5, 6, 7: Videos 4–6). Transplanted animals in the NSC group reached the maximum score (4.71 ± 0.22), which was significantly higher than the scores of the MSC group and control group (3.18 ± 0.40 and 2.59 ± 0.25, respectively).

The subscores for plantar stepping, coordination, paw position, trunk, and tail were tallied. At dpo 1, the subscore of animals in all groups decreased to zero. At dpo 28, the mean subscore of the NSC group was 1.07, a value that was significantly higher than the other groups (MSC group, 0.35; control group, 0.06) (Fig. 6b). Regarding changes in coordination, by dpo 28, 71% of the NSC-treated mice displayed some or a substantial restoration of coordination compared with 38% of the controls (Fig. 6c). Importantly, 29% of the NSC-treated mice were able to perform a mild trunk dysfunction, in contrast to 0% of the controls and 11% of the MSC group (Fig. 6d). Although the NSC group had a higher stepping score on the left and right sides compared with the MSC and control group, statistically significant differences were not observed among these groups (Fig. 6e, f). Thus, the hiPSC-NSC treatment was highly beneficial for the recovery of systemic function (e.g., coordination and trunk function) in the SCI mice.

## Discussion

Spinal cord injury (SCI) is currently the most difficult traumatic neurological condition to treat in the clinic. Following the primary injury, which causes immediate structural damage, a series of secondary injuries, including haemorrhage, enema, demyelination, and axonal and neuronal necrosis, occur [31]. Cell transplantation is a promising treatment, including NSCs/NPCs [32], MSCs [33], and Schwann cell-like cells [34]. To date, three main sources of NSCs have been described: direct isolation from the primary CNS tissue (either from the foetal or adult brain), differentiation from pluripotent stem cells, and transdifferentiation from somatic cells. Notably, iPSC-derived NSCs have striking advantages over ESC-derived NSCs, namely, the possibility of autologous transplantation [35]. Recent investigations have also examined the utility of MSCs recovered from tissues other than the bone marrow, including the umbilical cord and adipose tissue [36]. In the present study, we transplanted hiPSC-NSCs or huMSCs into the spinal cord following SCI.

The fibrous glial scar formed by infiltrated inflammatory cells (including microglia, fibroblasts, and reactive astrocytes) limits axon regeneration across the lesion [37, 38]. The scar tissue not only exerts a protective effect by limiting the spread of inflammation and secondary damage to adjacent intact tissue but also serves as an inhibitory barrier for axon regeneration [39, 40]. Most studies conducted to date have focused on the glial scar component of the scar tissue, while the fibrotic component has received less attention. In the present study, hiPSC-NSC and huMSC transplantation reduced spinal cord fibrosis. The hiPSC-NSCs were more effective, although the difference between hiPSC-NSCs and huMSCs was not significant. Fibrotic components are not expressed at high levels in the normal adult spinal cord, and previous studies have suggested that the excess deposition observed after SCI might be attributed to various sources, such as reactive astrocytes, macrophages, and fibroblasts [39, 41]. The fibrotic changes among the different groups were similar to changes in the GFAP levels, with the lowest levels observed in the hiPSC-NSC group.

The fate of transplanted cells is determined by the environment into which the cells are transplanted rather than the intrinsic properties of the cells. In our study, allogenic MSCs survived in injured spinal cords and integrated into the host tissue without the requirement for administering immunosuppressive agents. In the present study, the transplanted huMSCs were positive for GFAP immunostaining at dpo 14, thus confirming the differentiation of huMSCs into the astrocytic lineage. The huMSCs have been confirmed to differentiate into CNS cell lineages in a rat SCI model [42]. Some authors investigated BM-MSC transplantation in dogs [43, 44]. Among them, Ryu et al. [43] recorded improved neurological outcomes in MSC groups after acute transplantation (1 week after trauma), since all dogs exhibited purposeful hind limb motion. Moreover, some MSCs expressed markers for neurons (NF160), neuronal nuclei (NeuN), and astrocytes (GFAP) [13]. Based on our data, the transplanted hiPSC-NSCs differentiated into NeuN- and β-tubulin isotype III (β III tubulin)-positive neurons and GFAP-positive astrocytes. The most common approach for stem cell transplantation in mouse models is to inject the cells into the epicentre of the injured site. However, the tissue environment of the injured CNS is unfavourable for graft cell survival [14, 45, 46]. The number of transplanted cells gradually decreases and cells disappear from the spinal cord 3 weeks after injection [9]. When the NSCs or MSCs were transplanted in the acute phase after injury, the stem cells were no longer detected at dpo 28.

SCI induces an increase in the expression of pro-inflammatory cytokines (TNF-α, IL-6, and IL-1β), while it decreases the expression of the anti-inflammatory cytokine IL-10 [47]. As shown in the study by Cheng et al. [5], the NSC medium was able to suppress the expression of the pro-inflammatory cytokine IL-6. In the present study, NSC transplantation decreased the IL-6 level, which promoted recovery and improved locomotion. However, the level of IL-6 in the MSC group was similar to the control group. In the course of healing from the injury, the VEGF level detected in the NSC group at dpo 28 was lower than at dpo 14. A significant difference in serum TNF-α levels was not observed among the different groups at different time points. In mouse models of SCI, TNF-α-expressing cells were initially observed around the injured site at 30 to 45 min after injury, and TNF-α expression was substantially increased from 3 to 24 h [48, 49]. In our study, TNF-α was initially detected at dpo 7, and thus we may have missed the change in its expression. Pro-inflammatory cytokines are released from damaged cells; reactive microglia then recruit immune cells from broken blood vessels and promote tissue repair. Because reactive microglia and recruited macrophages are the main inflammatory cytokine-producing cells, the inflammatory response is then further exacerbated. The administration of IL-6, which is an important trigger of inflammatory cytokine production, to the injured spinal cord causes increased recruitment of neutrophils, an expansion of the areas occupied by macrophages and activated microglia, and reduced axonal regeneration [50, 51]. Therefore, inflammatory cytokines are strongly associated with the secondary damage after SCI.

Immunofluorescence staining showed that hNSCs and huMSCs differentiate into neurons and astrocytes; however, the functional outcome differed between the NSC and the MSC group, and the NSCs produced a better outcome than PBS and MSCs. The results are consistent with the morphological changes. A positive effect of the huMSC treatment on function was observed, but the difference was not statistically significant. The results differed from other studies [52, 53]. These opposing results are potentially attributed to the time of transplantation. Moreover, hiPSC-NSCs promoted functional recovery in SCI-induce mice not only primarily through cell differentiation and direct replacement of the lost cells but also through neuroprotective mechanisms, such as immunomodulatory factors to enhance axonal growth, modulate the environment, and reduce neuroinflammation [22].

## Conclusions

Based on our results, hiPSC-NSCs are capable of surviving and differentiating in an injured spinal cord. hiPSC-NSCs reduced the levels of pro-inflammatory cytokines after SCI and hence subsequently alleviated secondary damage, as evidenced by the reductions in glial scar and fibrotic scar formation. Therefore, hiPSC-NSC transplantation for acute SCI promotes the recovery of limb function, indicating that hiPSC-NSCs are a promising cell type for SCI treatment.

## Supplementary Information

**Additional file 1: Table 1.** The detail information of antibodies.

**Additional file 2: Video 1.** The video of mouse in control group at dpo1.

**Additional file 3: Video 2.** The video of mouse in NSC group at dpo1.

**Additional file 4: Video 3.** The video of mouse in MSC group at dpo1.

**Additional file 5: Video 4.** The video of mouse in control group at dpo28.

**Additional file 6: Video 5.** The video of mouse in NSC group at dpo28.

**Additional file 7: Video 6.** The video of mouse in MSC group at dpo28.

## Data Availability

The data that support the findings of this study are available from the corresponding author upon reasonable request.

## Abbreviations

SCI: Spinal cord injury
PBS: Phosphate-buffered saline
ESCs: Embryonic stem cells
iPSCs: Induced pluripotent stem cells
MSCs: Mesenchymal stem cells
NSCs: Neural stem cells
hiPSC-NSCs: Human iPSC-derived NSCs
huMSCs: Human umbilical cord mesenchymal stem cells
HE: Haematoxylin-eosin
SOX2: SRY-related high-mobility-group (HMG)-box protein-2
PAX6: Paired box 6
OCT4: Octamer binding transcription factor 4
CD: Cluster of differentiation
GFAP: Glial fibrillary acidic protein
BMS: Basso Mouse Scale
HuNu: Human nuclei
VEGF: Vascular endothelial growth factor
IL-6: Interleukin-6
TNF-α: Tumour necrosis factor-α
