# Supplementary material for: hiPSC-derived NSCs effectively promote the functional recovery of acute spinal cord injury in mice
Source: Stem Cell Res Ther. 2021 Mar 11;12:172. doi: 10.1186/s13287-021-02217-9 (PMC7953804; doi:10.1186/s13287-021-02217-9)
Supplement: Supplementary file 1 — Additional file 1: Table 1. The detail information of antibodies. [file 13287_2021_2217_MOESM1_ESM.doc]

Supplement

Table1. The detail information of antibodies

| **Name** | **Dilution/**  **Concentration** | **Company Cat # and RRID** |
| --- | --- | --- |
| Rabbit anti-OCT4 | 1:200 | Abcam Cat# ab18976, RRID:AB_444714 |
| Mouse anti-NESTIN | 1:200 | Abcam Cat# ab6320, RRID:AB_308832 |
| Anti-SOX2 antibody | 1:500 | Abcam Cat# ab97959, RRID:AB_2341193 |
| Mouse anti-PAX6 | 1:200 | Abcam Cat# ab78545, RRID:AB_1566562 |
| Nuclei antibody [235-1] | 1:200 | GeneTex Cat# GTX82624, RRID:AB_11162242 |
| GFAP Polyclonal Antibody | 1:300 | Thermo Fisher Scientific Cat# PA5-85109, RRID:AB_2792257 |
| Neuron specific beta III Tubulin antibody - Neuronal Marker | 1:300 | Abcam Cat# ab18207, RRID:AB_444319 |
| Goat anti-Rabbit IgG (H+L) Cross-Adsorbed Secondary Antibody, FITC | 1:100 | Thermo Fisher Scientific Cat# F-2765, RRID:AB_2536525 |
| Goat anti-Mouse IgG (H+L) Secondary Antibody, DyLight 594 | 1:200 | Thermo Fisher Scientific Cat# 35510, RRID:AB_1185569 |
| ProLong® Gold Antifade Reagent with DAPI #8961 | —— | Cell signaling, Cat#8961 |
